# Role of p66shc in skeletal muscle function

**DOI:** 10.1038/s41598-017-06363-0

**Published:** 2017-07-24

**Authors:** Veronica Granatiero, Gaia Gherardi, Matteo Vianello, Elsa Salerno, Erika Zecchini, Luana Toniolo, Giorgia Pallafacchina, Marta Murgia, Bert Blaauw, Rosario Rizzuto, Cristina Mammucari

**Affiliations:** 1 Department of Biomedical Sciences, University of Padua, Padua, Italy; 2 CNR Neuroscience Institute, Padua, Italy; 3 Venetian Institute of Molecular Medicine (VIMM), Padua, Italy; 4 Present Address: Weill Cornell Medical College, New York City, NY USA

## Abstract

p66shc is a growth factor adaptor protein that contributes to mitochondrial ROS production. p66shc is involved in insulin signaling and its deletion exerts a protective effect against diet-induced obesity. In light of the role of skeletal muscle activity in the control of systemic metabolism and obesity, we investigated which is the contribution of p66shc in regulating muscle structure and function. Here, we show that p66shc^−/−^ muscles are undistinguishable from controls in terms of size, resistance to denervation-induced atrophy, and force. However, p66shc^−/−^ mice perform slightly better than wild type animals during repetitive downhill running. Analysis of the effects after placing mice on a high fat diet (HFD) regimen demonstrated that running distance is greatly reduced in obese wild type animals, but not in overweight-resistant p66shc^−/−^ mice. In addition, muscle force measured after exercise decreases upon HFD in wild type mice while p66shc^−/−^ animals are protected. Our data indicate that p66shc affect the response to damage of adult muscle in chow diet, and it determines the maintenance of muscle force and exercise performance upon a HFD regimen.

## Introduction

The widely recognized dual role of ROS, indispensable signaling molecules in physiological settings and detrimental factors in pathological conditions, has been documented in a variety of tissues including skeletal muscle. ROS play a physiological signaling role in the adaptation to exercise^1, 2^, while muscle fatigue is due to production of damaging amounts of ROS. Moreover, increased ROS production and activation of antioxidant systems have been observed in conditions of muscle atrophy^3^. However, whether a causal link exists between ROS synthesis and atrophy induction is still debated. In an animal model of mechanical ventilation, antioxidant treatment successfully reduced atrophy, indicating that ROS actively contributes to muscle loss^3^. On the other hand, in a different atrophy model, i.e. short-term hind limb suspension, atrophy was not prevented by antioxidant treatment^4^.

p66shc is the 66 kDa protein codified by the shc locus of growth factors adaptors^5^. It translocates to the mitochondrial matrix in response to oxidative stress, via a mechanism that implies phosphorylation by protein kinase C β and recognition by the prolyl isomerase Pin1^6^. Once imported, mitochondrial p66shc generates ROS which induce apoptosis^7^. Inactivation of p66shc has been reported to exert protection against stress-related insults in different tissues. For example, in the heart p66shc ablation protects from ischemia-reperfusion injury by preventing oxidative stress^8^. Protection from tissue damage and accelerated muscle regeneration in p66shc^−/−^ mice has also been observed after hind limb ischemia^9^.

p66shc expression and consequently ROS production are upregulated by hyperglycemia in different cell models^10–13^. In adipocytes, insulin stimulates p66shc-dependent ROS production, which in turn regulates insulin signaling, sustaining triglyceride accumulation and obesity. p66shc^−/−^ mice are resistant to diet-induced obesity and have increased metabolic rate^14^. p66shc^−/−^ mice are also more insulin sensitive and glucose tolerant compared to wild type animals^15^. In addition, beneficial effects of p66shc deletion on diabetes-related complications have been reported^16–19^. However, a detailed analysis of the p66shc^−/−^ mouse model revealed a fourfold increase in the expression of p46shc, the 46 kDa isoform encoded by the shc locus. Comparison with a “pure” p66shc^−/−^ mouse strain suggested that, while p66shc deletion accounts for the increased insulin sensitivity and glucose tolerance, resistance to diet-induced obesity may be due to p46shc overexpression in adipose tissue^15^. Protection from obesity was reported also in p66shc^−/−^ lep^Ob/Ob^ mice, a genetic model of obesity^20^, even though adipose tissue analysis and metabolic parameters demonstrated that p66shc deletion does not protect lep^Ob/Ob^ mice from glucose intolerance and insulin resistance^21^, suggesting a different role of p66shc in different obesity models.

Together with liver and fat, skeletal muscle greatly affects organism metabolism. Muscle activity and exercise contribute to the maintenance of glucose tolerance, while inactivity is one of the major risk factors of metabolic syndrome development, type 2 diabetes and subsequent complications^22, 23^. p66shc regulates glucose transport in skeletal muscle myoblasts, by modulating the expression of glucose trasporters GLUT1 and GLUT3. In particular, expression of antisense p66shc is sufficient to induce GLUT1 and GLUT3 expression, while overexpression of p66shc causes a reduction of transporters expression and of glucose uptake rate^24^. Studies on the association of exercise with HFD highlighted the positive effect of training to HFD-induced insulin resistance and impairment of glucose uptake, although the scenario remains complex, especially when translated to humans^25, 26^. Mitochondria are undoubtedly the main source of ROS in skeletal muscle, and acute exercise has been shown to trigger increased p66shc expression and H_2_O_2_ content^27^. However, whether p66shc-induced mitochondrial ROS (mROS) production affects skeletal muscle homeostasis remains to be determined.

In this manuscript, we aimed to determine whether p66shc deletion affects adult skeletal muscle structure and function, both in chow and in HFD, and to clarify whether skeletal muscle contributes to the p66shc^−/−^ metabolic phenotype. Our results suggest that p66shc is required to maintain performance during downhill running in chow diet, while its absence exerts a protective effect against the decrease in running distance and muscle force caused by HFD.

## Results

### Skeletal muscle fiber size and myosin composition are unaffected by p66shc deletion

**Figure 1:**
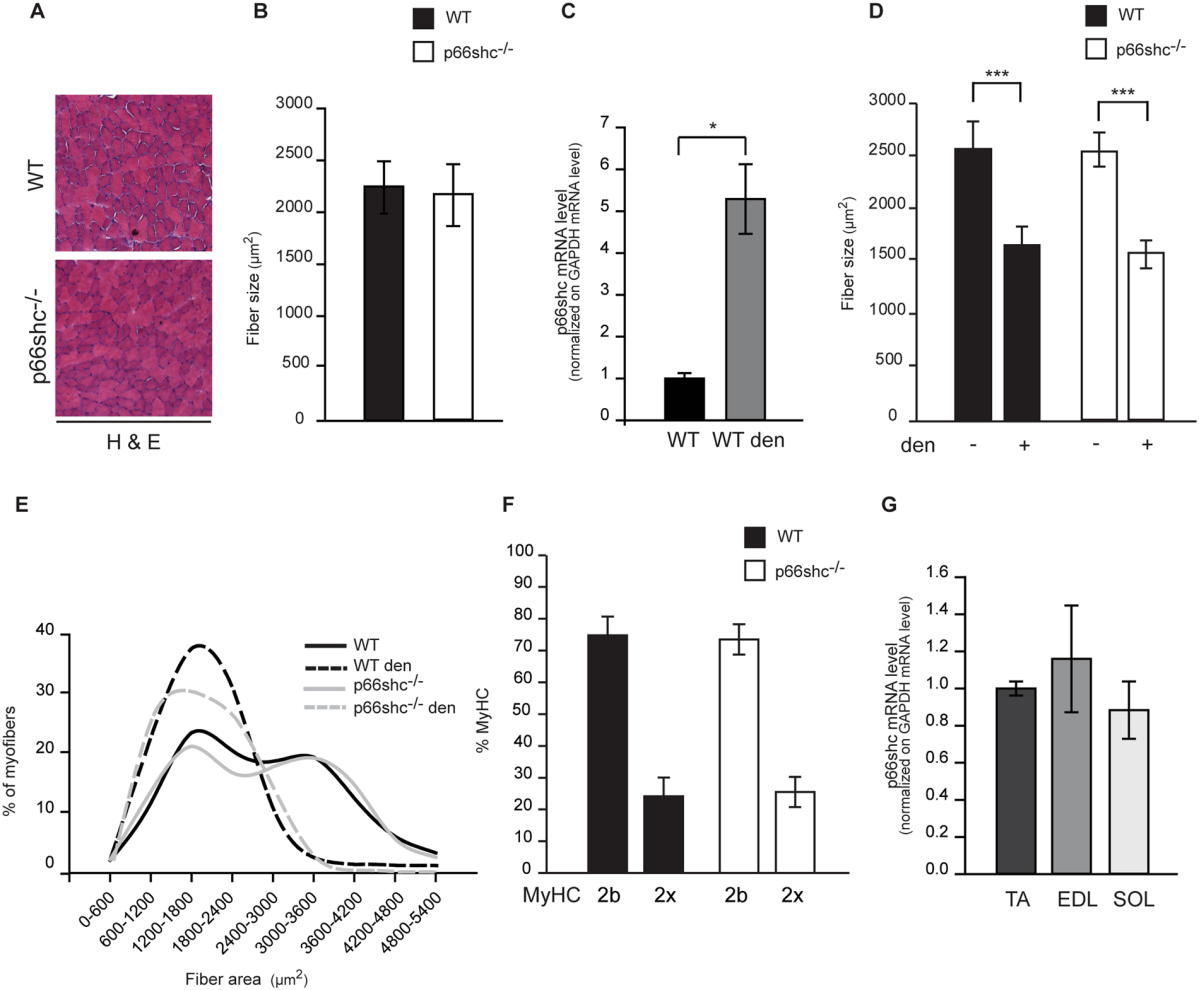
Skeletal muscle fiber size and myosin composition are unaffected by p66shc deletion. (**A**) Hematoxylin and Eosin (H&E) staining of transversal cryosections of wt and p66shc^−/−^ TA muscles. (**B**) Mean fiber size of TA muscles of wt and p66shc^−/−^ mice. Data are presented as mean ± SEM (3 animals per group). (**C**) mRNA expression levels of p66shc in wt and in denervated (den) TA muscles. GAPDH was used as control. *p < 0.05, t-test (Mann-Whitney rank sum test, two-tailed, unpaired) of 4 animals per condition. Data are presented as mean ± SEM (**D**) Mean fiber size of TA muscles of denervated (den) or not wt and p66shc^−/−^ mice. ***p < 0.005, pairwise multiple comparison (Holm-Sidak method) of 3 animals per condition. Data are presented as mean ± SEM. (**E**) Fiber size distribution of TA muscles of wt and p66shc^−/−^ mice. Data are presented as mean ± SEM (3 animals per group). (**F**) Quantitative myosin distribution analysis of wt and p66shc^−/−^ muscle mice. Data are presented as mean ± SEM (3 animals per group). (**G**) mRNA expression levels of p66shc in TA, EDL and soleus (SOL) muscles. GAPDH was used as control. Data are presented as mean ± SEM (5 animals per group).

To carry out a comprehensive study of the role of p66shc-induced mROS on skeletal muscle homeostasis, we analyzed structure and function of adult muscles of total p66shc^−/−^ mice^5^. After performing a Hematoxylin/Eosin (H&E) staining of transversal cryosections, which detected a normal structure in p66shc^−/−^ muscles **(**Fig. 1A **)**, we asked whether p66shc deletion affects muscle trophism in resting conditions. Fiber size measurements of tibialis anterior (TA) muscles did not reveal any difference between p66shc^−/−^ and wild type (wt) animals **(**Fig. 1B **)**, indicating that p66shc does not contribute to healthy muscle size. However, whether reduction in mROS production by p66shc deletion prevents muscle loss is still unclear. Indeed, the role of ROS in muscle atrophy is controversial and greatly depends on the etiology of muscle loss^3^. To clarify this issue, we used a denervation-induced model of muscle atrophy. p66shc mRNA levels were greatly increased by denervation **(**Fig. 1C **)**, indicative of a denervation-induced redox response. However, fiber size analysis, performed 12 days after cutting the sciatic nerve, demonstrated no differences in size between p66shc^−/−^ and wt hind limb muscles **(**Fig. 1D **)**, despite subtle differences in fiber size distribution **(**Fig. 1E **)**. This result indicates that p66shc does not regulate fiber size, neither in normal conditions nor in denervation-induced atrophy. Still we wondered whether p66shc deletion modifies muscle contractile properties and metabolism. Muscles are composed of different fiber types, according to their myosin composition and metabolic features. Myosins are classified from slow to fast according to the scheme 1-2A-2X-2B and myosin composition determines the contractile properties of a fiber^28^. In addition, fibers are also classified based on their metabolic properties, ranging from purely glycolytic to mitochondria-rich oxidative fibers. We evaluated all these parameters in p66shc^−/−^ hind limb muscles. Firstly, we performed an electrophoresis analysis in order to determine myosin composition of gastrocnemius muscles **(**Fig. 1F **)**. Then, we measured p66shc expression levels in muscles with different metabolic properties, ranging from glycolytic (EDL) to oxidative (soleus) muscles **(**Fig. 1G **)**. In neither case we observed differences between wt and p66shc^−/−^ muscles.

**Figure 2:**
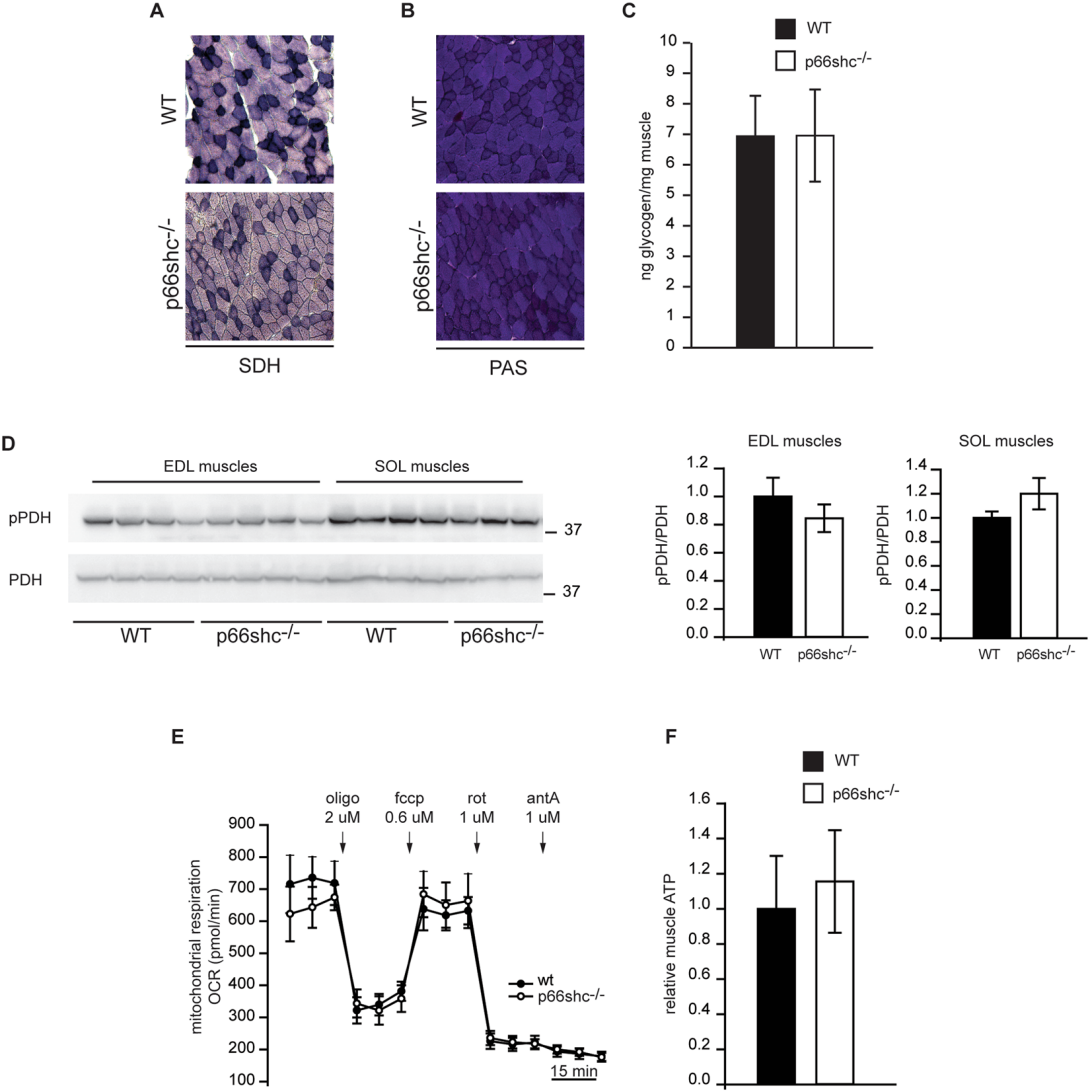
Metabolic parameters of p66shc^−/−^ muscles. (**A**) Succinyl-Dehydrogenase (SDH) activity assay of wt and p66shc^−/−^ TA muscles. (**B**) Periodic Acid–Schiff (PAS) staining for glycogen content. (**C**) Glycogen quantification of wt and p66shc^−/−^ muscles mice. Data are presented as mean ± SEM (3 animals per group). (**D**) Phosphorylation levels of PDH in EDL and soleus (SOL) muscles and quantification by densitometry. Data are presented as mean ± SEM (5 animals per group). Cropped blots are displayed, and full-length blots are presented in Supplementary Figure S1. (**E**) OCR rate measurements in adult myofibers. Data are presented as mean ± SEM (3 animals per group). (**F**) ATP measurements in muscle homogenates. Data are presented as mean ± SEM (5 animals per group).

Next, we investigated whether p66shc deletion affects fiber oxidative metabolism. We discerned glycolytic and oxidative fibers on the basis of a colorimetric assay for the activity of the mitochondrial enzyme succinyl-dehydrogenase (SDH). The proportion of glycolytic versus oxidative fibers was unaffected in p66shc^−/−^ TA muscles, indicating that p66shc does not determine a metabolic shift of muscle fibers **(**Fig. 2A **)**. To clarify whether p66shc regulates glucose metabolism of healthy muscles, intracellular glycogen content was evaluated. In both Periodic Acid-Schiff (PAS) staining and glycogen level measurements, no differences were observed between wt and p66shc^−/−^ muscles **(**Fig. 2B,C **)**. In order to understand whether mitochondrial function was affected by p66shc deletion, we measured pyruvate dehydrogenase (PDH) phosphorylation levels **(**Fig. 2D **)**, which are in inverse correlation with the activity status of PDH. In addition, we measured Oxygen Consumption Rate (OCR) in single isolated myofibers **(**Fig. 2E **)** and ATP production **(**Fig. 2F **)**. Our data demonstrate that deletion of p66shc does not impinge on basic mitochondrial functions.

Altogether these data demonstrate that p66shc does not contribute to muscle homeostasis of healthy animals, neither in terms of fiber size nor of fiber type and metabolism. Moreover, p66shc does not play a fundamental role in denervation-induced atrophy.

### p66shc^−/−^ mice exhibit unaltered endurance exercise performance but perform slightly better during downhill running

**Figure 3:**
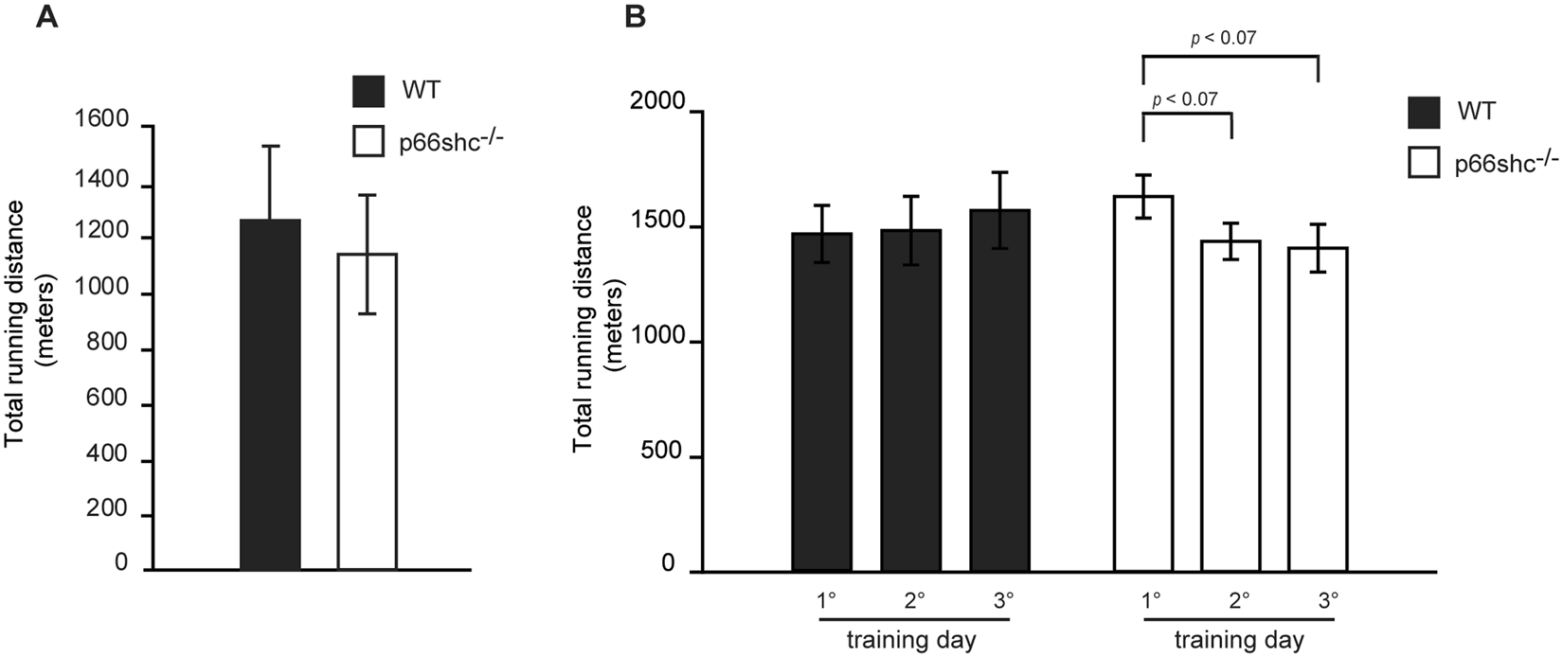
p66shc deletion negatively affects downhill exercise performance. (**A**) Mean maximal running distance of a single bout of run on a treadmill of wt and p66shc^−/−^ mice. Data are presented as mean ± SEM (8 animals per group). (**B**) Mean maximal running distances after each of 3 days of downhill run on a treadmill of wt and p66shc^−/−^ mice. p < 0.07, pairwise multiple comparison (Holm-Sidak method) of 5 animals per condition. Data are presented as mean ± SEM.

Whether exercise-induced ROS production causes muscle damage and contributes to muscle fatigue is still debated. Several lines of evidence suggest that this is not always the case. For example, in specific settings, treatment with antioxidants reduces exercise performance^2, 29^. We asked whether p66shc-dependent ROS production contributes to exercise performance. To answer this question, we decided to compare maximal running distance and muscle force of p66shc^−/−^ and wt mice. Exhaustion exercise performance was evaluated by a single bout of run on a treadmill. Wt and p66shc^−/−^ mice performed similarly in terms of running distance **(**Fig. 3A **)**. However, the beneficial effects of ROS on exercise adaptation are particularly evident during repeated bouts of damaging eccentric exercise^2^. Thus, we hypothesized that p66shc deletion would blunt this effect, causing a progressive loss of muscle performance, as occurs in anti-oxidant treated mice^2^. Accordingly, experiments of exhaustive downhill running were performed in three consecutive days and running distances were recorded. p66shc deletion caused an almost significant reduction in running performance, indicating that p66shc-dependent ROS production is instrumental for muscle recovery upon damage (Fig. 3B).

### Trophic and metabolic properties of p66shc^−/−^ muscles upon HFD

**Figure 4:**
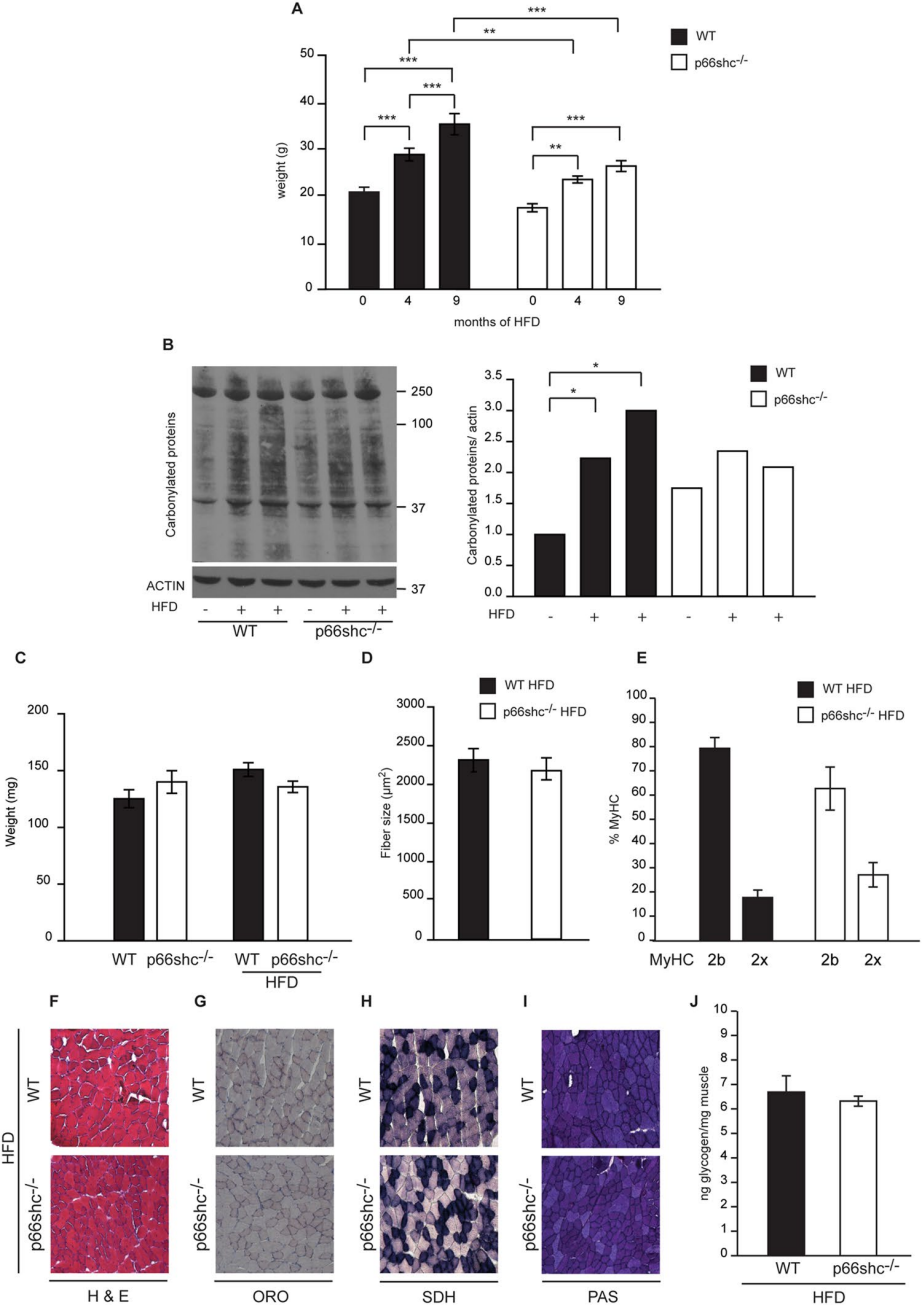
Trophic and metabolic properties of p66shc^−/−^ muscles upon HFD. (**A**) Female wt and p66shc^−/−^ mice were fed with HFD for 4 and 9 months and body weights were plotted. **p < 0.01, ***p < 0.005, pairwise multiple comparison (Holm-Sidak method) of 7 animals per condition. Data are presented as mean ± SEM. (**B**) Western blot analysis of carbonylated proteins of wt and p66shc^−/−^ muscles mice after HFD (9 mo) and quantification by densitometry. *p < 0.05 pairwise multiple comparison (Holm-Sidak method). Cropped blots are displayed, and full-length blots are presented in Supplementary Figure S1. (**C**) Gastrocnemius muscle weights of wt and p66shc^−/−^ mice in chow and in HFD conditions. Data are presented as mean ± SEM (5 animals per group). (**D**) Mean fiber size of TA muscles of wt and p66shc^−/−^ mice after HFD (9 mo). Data are presented as mean ± SEM (3 animals per group).(**E**) Quantitative myosin distribution analysis of wt and p66shc^−/−^ muscles mice after HFD (9 mo). Data are presented as mean ± SEM (5 animals per group). (**F**) H&E staining of transversal cryosections of TA muscles after HFD (9 mo). (**G**–**J**) Analysis of the metabolic properties of wt or p66shc^−/−^ muscles after HFD (9 mo). (**G**) Lipid accumulation detected by Oil-red-O (ORO) staining. (**H**) SDH activity assay. (**I**) Glycogen amount detected by Periodic Acid–Schiff (PAS) staining. (**J**) Glycogen amount quantification Data are presented as mean ± SEM (5 animals per group).

Increased oxidative stress is both a trigger and a consequence of obesity and antioxidant treatment improves metabolic parameters^30^. It has been demonstrated that p66shc^−/−^ mice are protected from HFD-induced obesity^14, 15^. Similarly, protection by p66shc deletion was observed in lep^Ob/Ob^ mice^20^, although the beneficial effects on glucose intolerance and insulin resistance have been questioned^21^. Muscle atrophy and weakness have been linked to obesity^31, 32^. We wondered whether skeletal muscle fiber size, myosin composition and metabolic parameters are affected by an experimental HFD regimen in which 60% of the total energy derives from fat, and whether p66shc plays a role in this context. Female mice were fed with HFD for 4 and 9 months. As reported^14^, increased body weight was apparent in wt mice, while it was less pronounced in p66shc^−/−^ mice **(**Fig. 4A **)**. We investigated whether this HFD treatment triggers ROS production in skeletal muscle and whether p66shc is required. Accordingly, analysis of carbonylated proteins on muscle extracts was performed. HFD caused an increase in ROS production only in wt samples **(**Fig. 4B **)**, indicating that p66shc is required for HFD-induced ROS generation in skeletal muscle. However, muscle weight was similar between chow and HFD-fed mice, in both backgrounds **(**Fig. 4C **)**. Moreover, fiber size analysis showed no differences between chow (reported in Fig. 1B) and HFD-treated mice **(**Fig. 4D **)** in both genotypes. We also asked whether HFD regimen affects myosin composition, compared to data reported in Fig. 1F. Quantitative myosin analysis demonstrated that HFD does not affect the relative myosin distribution, neither in wt, nor in p66shc^−/−^ muscles **(**Fig. 4E **)**. We reasoned that subtler metabolic parameters could be affected. However, H&E staining of HFD muscle cryosections, both wt and p66shc^−/−^, were compatible with a healthy tissue structure **(**Fig. 4F **)**, although lipids visualization by Oil Red O (ORO) staining demonstrates lipid accumulation in interstitial spaces **(**Fig. 4G **)**. We monitored mitochondrial activity by SDH staining, without observing important differences between p66shc^−/−^ and wt muscles **(**Fig. 4H **)**. Lastly, qualitative and quantitative measurements of glycogen content (as reported in Fig. 2B,C) demonstrated that HFD regimen does not significantly affect this parameter **(**Fig. 4I–J **)**.

Overall these data demonstrate that, in our experimental conditions, HFD, independently of the contribution of p66shc-induced ROS, does not significantly alter trophic and metabolic properties of skeletal muscle.

### p66shc^−/−^ mice maintain muscle force and exercise performance on HFD regimen

**Figure 5:**
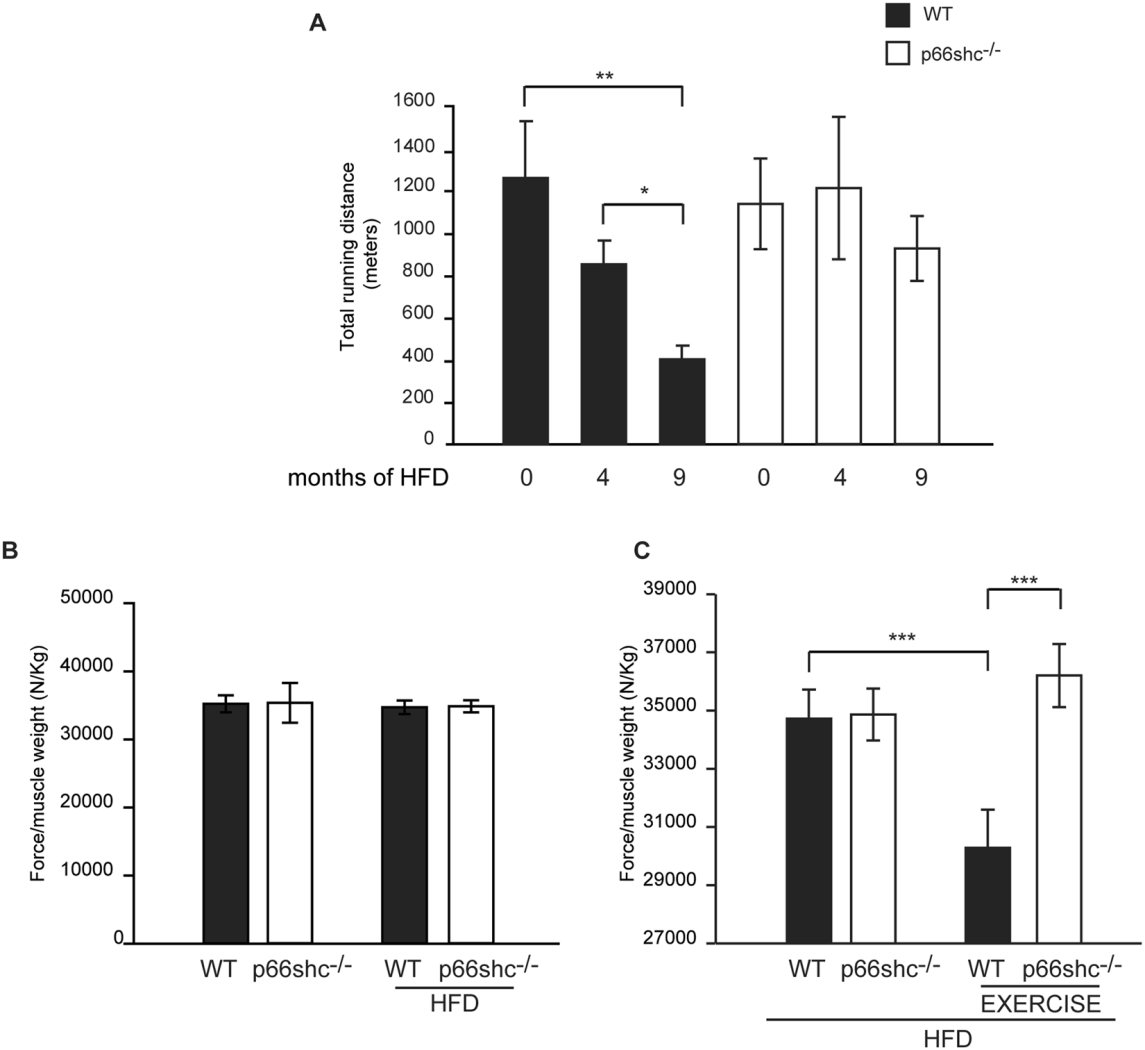
p66shc^−/−^ mice maintain muscle force and exercise performance on HFD regimen. (**A**) Mean maximal running times of wt and p66shc^−/−^ mice fed with HFD for 4 and 9 months after a single bout of run. *p < 0.05 **p < 0.01, pairwise multiple comparison (Holm-Sidak method) of 5 animals per condition. Data are presented as mean ± SEM. (**B**) Normalized maximal force production from gastrocnemius muscles of mice fed with chow diet or HFD for 9 months. Data are presented as mean ± SEM (7 animals per group). (**C**) Normalized maximal force production from gastrocnemius muscles of mice fed with HFD for 9 months before and after a treadmill run until exhaustion. ***p < 0.005, pairwise multiple comparison (Holm-Sidak method) of 7 animals per condition. Data are presented as mean ± SEM.

We wondered whether HFD-induced obesity affects exercise performance and force generation, and whether these are controlled by p66shc. Mice were subjected to a single bout of strenuous exercise on a treadmill and running distance was measured. As reported in Fig. 3A, wt animals on chow diet run about 1,200 meters. When kept in HFD for 4 months, running distance drastically decreased to about 870 meters, and after 9 months of HFD average running distance was only 400 meters **(**Fig. 5A **)**. On the contrary, p66shc^−/−^ mice, which showed a maximum average running distance of 1,200 meters in chow diet (see Fig. 3A), maintained similar running distance after 4 and 9 months of HFD **(**Fig. 5A **)**. In order to understand if the reduction in exercise performance in wt mice fed with HFD for 9 months could be explained, at least partially, by a reduction in muscle force, we first examined the effects of the diet on tetanic strength. HFD did not affect gastrocnemius tetanic force production of sedentary mice *in vivo* **(**Fig. 5B **)**. However, when force was measured immediately after one bout of strenuous exercise, wt mice showed a marked reduction **(**Fig. 5C **)**. Interestingly, despite the fact that p66shc^−/−^ mice run significantly further, they didn’t show any reduction in normalized force after a treadmill run until exhaustion.

These results suggest that diet-induced obesity affects both the performance during resistance training and the muscle force generation after exercise, and that p66shc deletion exerts a protective effect, both impinging on whole organism metabolism, and on skeletal muscle *per se*.

### Regulation of muscle autophagy and UPR markers by p66shc

**Figure 6:**
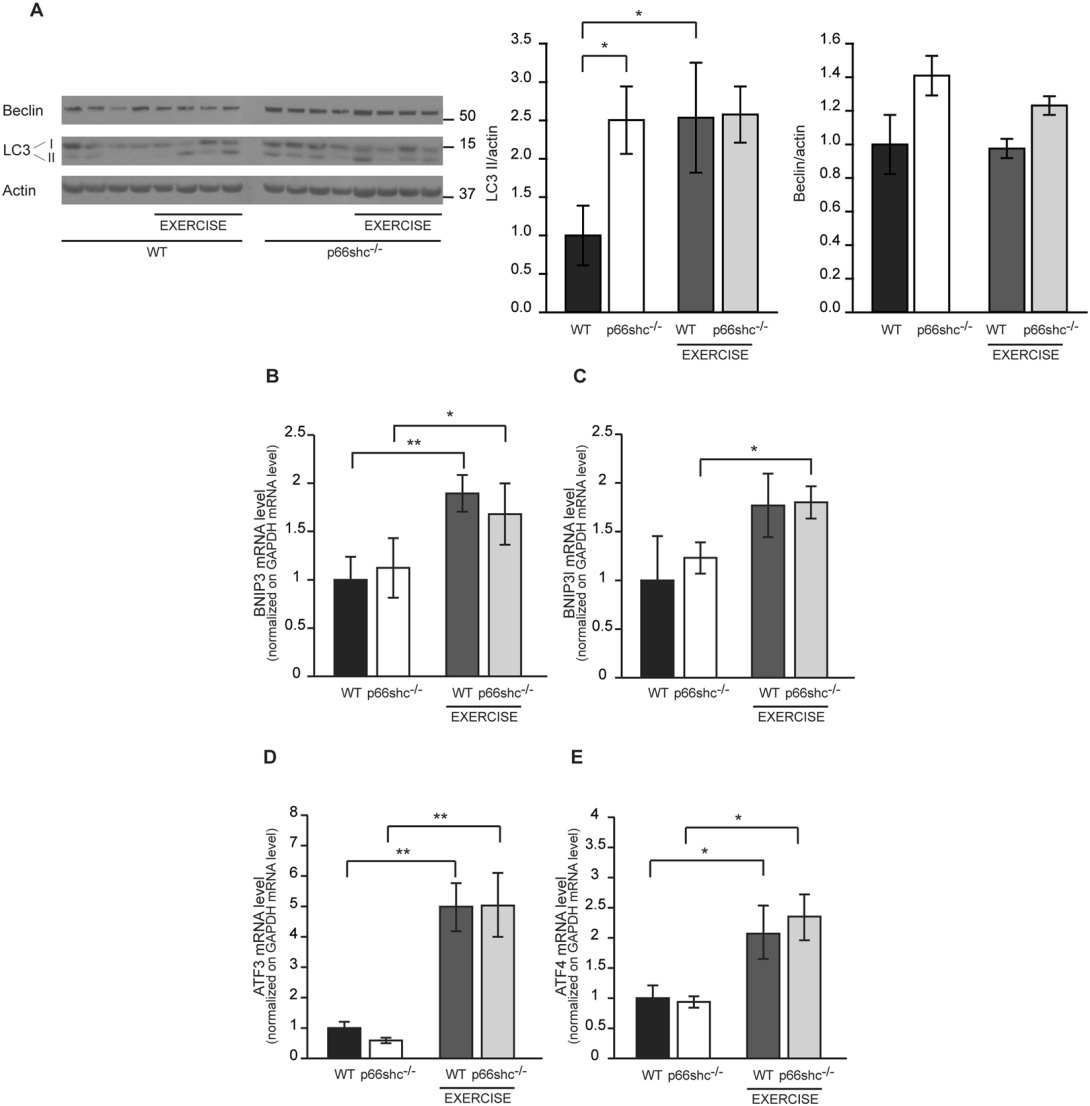
Muscle autophagy and ER stress are not regulated by p66shc in chow-diet treated mice. (**A**) Immunoblotting of gastrocnemius muscles of wt and p66shc^−/−^ mice before and immediately after (exercise) a single bout of exhaustion running and quantification by densitometry. *p < 0.05, pairwise multiple comparison (Holm-Sidak method) of 4 animals per condition. Data are presented as mean ± SEM. Cropped blots are displayed, and full-length blots are presented in Supplementary Figure S1. (**B**–**E**) qRT-PCR for Bnip3 (**B**), Bnip3l (**C**), ATF3 (**D**) and ATF4 (**E**) transcripts. Data are normalized on GAPDH mRNA level. *p < 0.05, **p < 0.01, pairwise multiple comparison (Holm-Sidak method) of 4 animals per condition. Data are presented as mean ± SEM.

The autophagy-lysosome system plays a major role in the maintenance of skeletal muscle homeostasis. A basal level of autophagy is constantly present in resting conditions, where it guarantees the removal of dysfunctional macromolecules and organelles. Physiologic and pathologic triggers are responsible for an increase of autophagy flux beyond the resting level. For example, muscle autophagy is induced by starvation^33^. Autophagy is also induced by exercise^34, 35^ and contributes to its beneficial metabolic adaptations, as demonstrated in a mouse model in which autophagy induction is prevented^35^. A detailed analysis of different exercise protocols demonstrated that muscle-specific inhibition of autophagy does not compromise running performance in a uphill treadmill exercise^2^. However, the same study demonstrated that muscle autophagy is required for the maintenance of mitochondrial function during damaging downhill exercise, which is characterized by increased ROS production^2^. Thus, we asked whether p66shc contributes to exercise-induced autophagy. Muscles were withdrawn immediately after a single bout of exhaustion running on a treadmill and protein and mRNA levels of autophagy markers were measured. As already reported^2, 34, 35^, also in our model exercise induced LC3B lipidation in wt muscles. A similar effect was observed in p66shc^−/−^ muscles **(**Fig. 6A **)**. In addition, although Beclin expression was mostly unaffected by the exercise protocol **(**Fig. 6A **)**, Bnip3 and Bnip3l transcription was induced by exercise both in wt and in p66shc^−/−^ muscles **(**Fig. 6B,C **)**. These data confirm the induction of autophagy upon exercise but do not support a role of p66shc in this process.

We then hypothesized that p66shc may play a role in the regulation of the unfolded protein response (UPR), which maintains endoplasmic reticulum (ER) homeostasis upon stress, and has been reported to contribute to adaptation of skeletal muscle upon exercise. Indeed, ER stress and UPR markers are induced upon a bout of exhaustive treadmill running^36^ and deletion of ATF6α, one of the UPR sensors^37^, hampers recovery from muscle damage upon repeated bouts of exhaustive exercise^36^. In agreement with previous data^36^, expression of ATF3 and ATF4, two ER stress markers, was induced in hind limb muscles upon one bout of exhaustive exercise, however no differences were observed between wt and p66shc^−/−^ muscles **(**Fig. 6D,E **)**.

In light of the positive effect of p66shc deletion on muscle performance and force in HFD, we wondered whether these two pathways, i.e. autophagy and UPR, are dysregulated in these conditions and whether p66shc contributes to their modulation.

**Figure 7:**
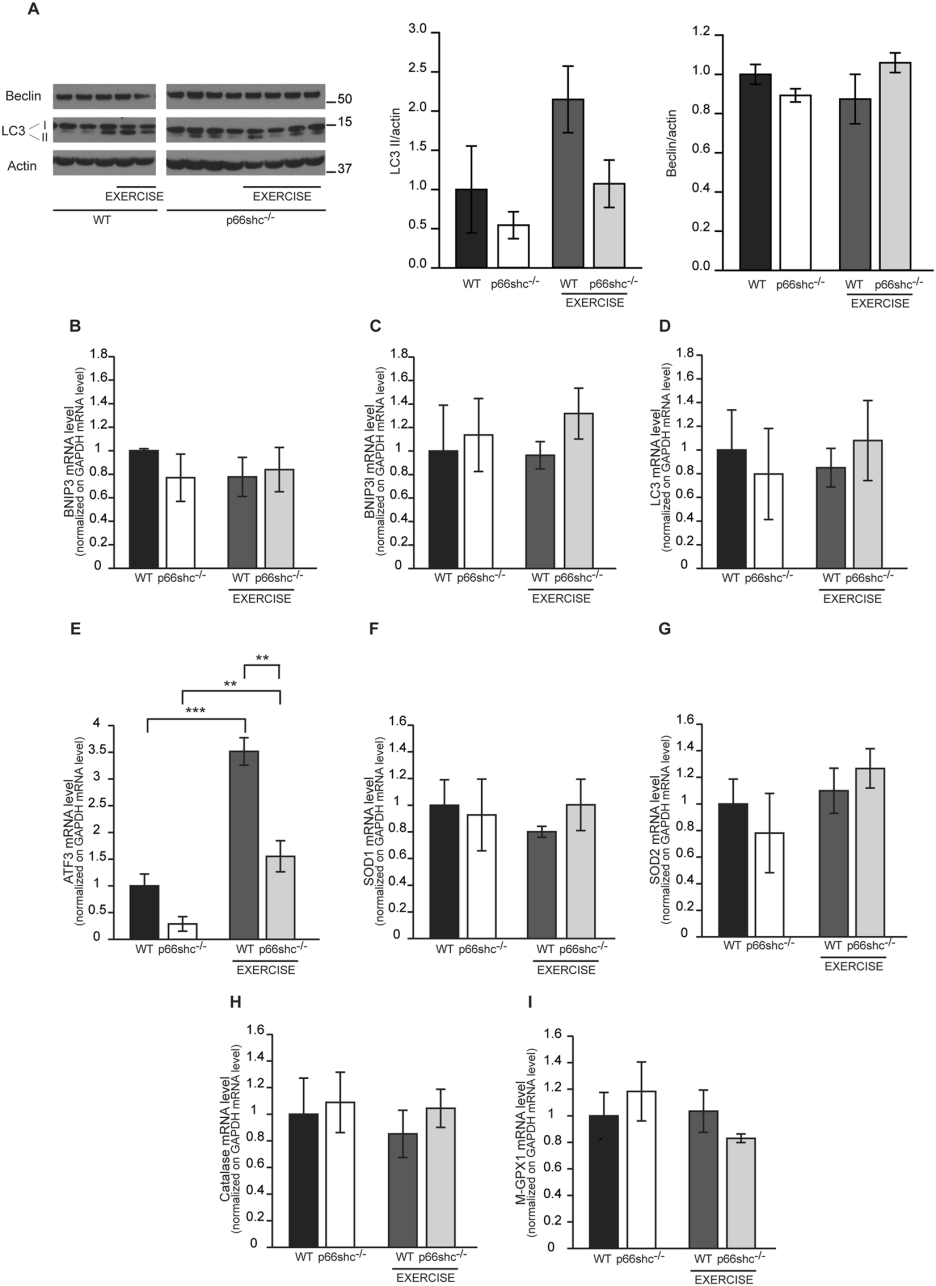
Analysis of signaling pathways in wt and p66shc^−/−^ HFD-treated mice. (**A**) Immunoblotting of muscles of wt and p66shc^−/−^ mice fed with HFD for 9 month before and immediately after (exercise) a single bout of exhaustion running and quantification by densitometry. Data are presented as mean ± SEM (3–4 animals per group). Cropped blots are displayed, and full-length blots are presented in Supplementary Figure S1. (**B**–**I**) qRT-PCR for Bnip3 (**B**), Bnip3l (**C**), LC3 (**D**), ATF3 (**E**), SOD1 (**F**), SOD2 (**G**), Catalase (**H**) and M-GPX1 (**I**) transcripts. Data are normalized on GAPDH mRNA level. **p < 0.01, ***p < 0.005, pairwise multiple comparison (Holm-Sidak method)of 4 animals per condition. Data are presented as mean ± SEM.

Analysis of LC3B lipidation demonstrated that autophagy is induced by exercise also in HFD-treated mice. However, protein levels of Beclin and mRNA expression levels of LC3, Bnip3 and Bnip3l were not significantly affected by exercise. In addition, p66shc deletion was not sufficient to restore autophagy gene expression upon exercise **(**Fig. 7A–D **)**. These data demonstrate that the differences in muscle force between HFD-fed p66shc^−/−^ and wt mice after exercise could not be explained by variations in autophagy modulation among the two genotypes.

Concerning ER stress and UPR response, an interesting indication came from the analysis of ATF3 expression. Indeed, as in chow diet, ATF3 was induced in wt muscles after exercise upon 9 months of HFD. However, the increase in ATF3 expression was less pronounced in p66shc^−/−^ muscles, suggesting a potential role of ER stress in the differential response to exercise of HFD treated p66shc compared to wt mice **(**Fig. 7E **)**.

Finally, basis on the importance that ROS play in muscle adaptation to exercise, we investigated the role of ROS scavengers. However, mRNA expression analysis confirmed the absence of a modulation of endogenous antioxidant systems by exercise in HFD, regardless of the genotype **(**Fig. 7F–I **)**.

## Discussion

ROS play a dual role in tissue homeostasis. At low, physiological concentrations, ROS act as positive and indispensable signaling molecules. On the other hand, dysregulated ROS production triggered by pathological stimuli causes damages to several cell structures which, if not adequately counteracted by ROS scavengers and repair enzymes, negatively affects cell function^38^. The boundary between these two opposite scenarios is not completely defined and, in specific settings, it is difficult to discern whether ROS are playing a beneficial or rather a damaging role. For example, in the case of skeletal muscle, the role of ROS is still controversial. If on one side the consensus is that damaging ROS production is increased by exercise and contributes to muscle fatigue, on the other hand the use of antioxidants has been proven to negatively affect muscle performance^1, 2^.

A significant amount of ROS originates as side product of mitochondrial respiration^39^. *In vivo* studies of mROS-related effects have been facilitated by the p66shc^−/−^ mouse model^5^. p66shc belongs to the shc family of adaptor proteins encoded by SHC1 gene which includes p66shc, p46shc and p52shc. Unique among the family, p66shc translocates to the mitochondrial matrix upon oxidative stress and induces mROS production^6^. Thus, the p66shc^−/−^ mouse appears to be an ideal model for the study of mROS in different organs and tissues and in various pathophysiological conditions. The most striking phenotype of p66shc^−/−^ mice is the fact that they are protected from HFD-induced obesity^14^.

We decided to explore whether p66shc deletion affects adult skeletal muscle structure and function, both in chow and in HFD in order to understand whether skeletal muscle contributes to the p66shc^−/−^ metabolic phenotype. In chow diet, p66shc appeared totally dispensable for the determination of muscle structure and metabolic properties, indicating that p66shc-dependent mROS do not play a major role in resting adult skeletal muscles. The response to denervation-induced atrophy was also unaffected by loss of p66shc. However, whether increased ROS contribute to muscle loss is not completely understood, and certainly depends on the atrophy model^3, 4^. We reasoned that p66shc-dependent mROS could still play a role during muscle activity. Running performance of p66shc^−/−^ mice during strenuous exercise was undistinguishable from wt animals, however, during downhill running, a condition that has been associated to ROS-induced muscle damage^40^, p66shc^−/−^ mice were slightly less fatigued than wt. Thus, despite the minor role of p66shc in resting adult skeletal muscle, its deletion has relevant effects in conditions of high levels of cell stress. The second aspect that we decided to investigate was the contribution of skeletal muscle to p66shc^−/−^ phenotype in HFD. p66shc^−/−^ mice are protected from diet-induced obesity and maintain glucose tolerance and insulin sensitivity^14, 15^. In agreement with previous reports, the HFD protocol used in our study caused obesity in wt animals but not in p66shc^−/−^ animals. Analysis of carbonylated proteins indicates increased ROS production by HFD, which is hampered in the absence of p66shc. However, a structural analysis of skeletal muscles upon HFD did not reveal substantial differences with chow-treated mice, neither in wt nor in p66shc^−/−^ mice, suggesting that induction of ROS within this HFD protocol is not sufficient to trigger atrophy and metabolic dysfunctions. While obese wt animals were characterized by impaired running ability, muscle performance of p66shc^−/−^ animals was unaffected. The most obvious reason could be the fact that p66shc^−/−^ mice were leaner. However, the direct contribution of skeletal muscle force to this result is demonstrated by the fact that measurements of normalized muscle strength after exercise revealed a decrease in tetanic force only in wt animals, while no reduction in force was observed in p66shc^−/−^ muscles.

Concerning the mechanism, our analysis of exercise-induced stress-related pathways, such as autophagy and UPR, did not reveal a strong connection with the observed phenotypes, indicating that further investigation is required.

Thus, our results indicate that the contribution of p66shc to muscle function becomes evident in conditions of maximal stress, like those occurring during strenuous exercise, while its role is dispensable for muscle homeostasis in resting conditions. A dual role is underlined: while in chow diet p66shc plays a protective role during downhill exercise, presumably contributing with essential ROS, in HFD the associated metabolic alterations prevail, and p66shc deletion protects muscles from loss of force and performance. Further studies will clarify the mechanism underlying these effects.

## Methods

### *In vivo* experiments

Procedures involving animals and their care were in accordance with the Italian law D. L.vo no 26/2014, and the experimental protocol was approved by the Ethical Committee of the University of Padova (Prot. n. 23 064).

The p66Shc^−/−^ mouse strain carries a targeted mutation of the Shc CH2 exon, as described in^5^. C57BL/6 J mice were used as wt controls.

Denervation was achieved by cutting the sciatic nerve high in the thigh. 12 days later histological analysis on muscle cryosections were performed as reported below.

For HFD experiments, after weaning animals were fed for 4 or 9 months with a diet in which 60% of the total energy derives from fat (Mucedola). Wt and p66Shc^*−/−*^ mice were randomly assigned to the HFD or to the standard diet group.

Exercise was performed by subjecting mice to a forced run on a treadmill device until exhaustion. Briefly, mice were first left to adapt to the treadmill for 5 min at a speed of 5 cm/sec. After this, exercise was performed by starting with a speed of 10 cm/sec that was increased with 2 cm/sec every 2 min until a maximum of 40 cm/sec. Once mice were unable to run for 5 seconds consecutively, they were removed from the treadmill. The eccentric training protocol consisted of 1 to 3 days of treadmill running to exhaustion, with a downhill 10° decline. After 15′ of adaptation from 7 to 17 cm/sec, exercise was performed by starting with a speed of 17 cm/sec that was increased with 2 cm/sec every 10 min until mice were unable to run for 5 seconds consecutively. Total running distance of each animal were recorded.

To measure muscle force in living animals, the contractile performance of gastrocnemius muscle *in vivo* was measured as described previously^41^. Briefly, anesthetized mice were placed on a thermostatically controlled table, keeping the knee stationary, and the foot firmly fixed to a footplate, which was connected to the shaft of the motor of a muscle-lever system (305B, Aurora Scientific). Contraction was elicited by electrical stimulation of sciatic nerve. Teflon-coated seven-stranded steel wires (AS 632, Cooner Sales) were implanted with sutures on either side of the sciatic nerve proximal to the knee before its branching. At the distal ends of the two wires, the insulation was removed, and the proximal ends were connected to a stimulator (S88, Grass). To avoid recruitment of the dorsal flexor muscles, the common peroneal nerve was cut.

### Histology and fluorescence microscopy

For fiber size measurements, 20 μm thick cryosections were fixed in 4% formaldehyde for 20 min, quenched with 50 mM NH_4_Cl in PBS and blocked in PBS containing 10% goat serum and 0.5% BSA for 20 min. Sections were then incubated with primary antibody Dystophin (Sigma) to label the sarcolemma for 1 h at 37 °C and washed 3 times in PBS. Alexa Fluor 488 conjugated secondary antibody (Life Technologies) was used. Fiber size measurements were performed with the Fiji distribution of ImageJ^42^. Mean fiber size of three muscles per group was calculated. SEM was calculated on the values of the mean fiber size of each muscle.

For Hematoxylin and Eosin (H&E) staining, 20 μm thick cryosections were stained using Rapid Frozen Sections H&E staining Kit (Bio-Optica) according to manufacturer’s instructions. Briefly, cryosections were incubated in hematoxylin solution for 60 seconds. Then, after 3 washes, they were incubated in eosin solution for 30 seconds. Finally, cryosections were dehydrated.

To detect SDH activity 20 μm thick cryosections were incubated in SDH solution (1 mg/mL of nitroblue tetrazolium, 0.2 M Na succinate in 0.2 M phosphate buffer, pH 7.4) for 20 min at 37 °C.

For glycogen content, 20 μm thick cryosections were stained using PAS staining system (Sigma) according to manufacturer’s instructions. Briefly, cryosections were incubated in Periodic acid solution for 5 min, washed, incubated in Schiff’s reagent for 15 min, washed again, and incubated in Gill’s hematoxylin solution for 2 min. Finally, after washing, cryosections were dehydrated.

To detect lipids content, Oil Red O (ORO) staining procedure was used as previously reported^43^. Briefly, cryosections were incubated in a freshly prepared Oil Red O working solution for 15 min. After being washed, they were incubated in hematoxylin solution for 30 seconds. Finally, after 3 washes, cryosections were dehydrated.

### Glycogen level measurement

Glycogen amount was measured by means of the colorimetric Glycogen Assay Kit II (Abcam) according to manufacturer’s instructions. Briefly, 20 mg of muscle tissue were homogenized in 250 µl of glycogen hydrolysis buffer. Samples were centrifuged a top speed for 5 min and the supernatant was collected. 2 µl of hydrolysis enzyme mix were added to 5 µl of samples in a 96 well plate. After an incubation of 30 min, reaction mix was added in each well. After 30 min of incubation, absorbance at 450 nm was measured.

### Electrophoresis for myosin heavy chain

Myosin heavy chain (MyHC) isoforms composition was determined as previously described^41^. Briefly, for separation of myosin heavy chain (MyHC) isoforms by electrophoresis, muscle samples were solubilized in Laemmli solution (62.5 mM Tris, pH 6.8; 10% glycerol, 2.3% SDS, and 5% β-mercaptoethanol), with 0.1% E-64 and 0.1% leupeptin (Sigma-Aldrich Corp., St Louis, MO, USA) as antiproteolytic factors. Samples were analyzed on 8% polyacrylamide slab gels after denaturation in SDS with a procedure derived from Blough *et al*.^44^, modifying stacking gel composition with 29% glycerol. Slabs 18 cm wide, 16 cm high, and 1 mm thick were used. Electrophoresis was run for 46 h, at 100 V for 3 h, and 230 V for the remaining time at 4 °C. Gels were silver stained (BioRad Silver Stain Plus, BioRad, Hercules, CA, USA). Four MyHC bands were separated in the region of 200 kDa, corresponding, in order of migration rate, from the fastest to the slowest.

### Gene Expression Analysis

Muscle RNA was prepared with the Promega SV Total RNA Isolation kit. cDNA was generated with SuperScript II reverse transcriptase (Life Technologies) and qRT-PCR was performed with iQ SYBR Green Supermix (Bio-rad). The primers were designed and analyzed with Primer3^45^. The housekeeping gene GAPDH were used as an internal control for cDNA quantification and normalization of the amplified products.

Real-time PCR primer sequences were the following:

p66Shc-fw: 5′ - CTGGAGGAAGGGGCTTCT-3′; p66Shc-rv: 5′-AGGCAGAGGAGGCAGGAT-3′;

GAPDH-fw: 5′- CACCATCTTCCAGGAGCGAG-3′; GAPDH-rv: 5′- CCTTCTCCATGGTGGTGAAGAC-3′;

Bnip3-fw: 5′- GTTCCAGCCTCGGTTTCTATT-3′; Bnip3-rv: 5′- TTCTCTCCAATGCTATGGGTATC-3′;

Bnip3l-fw: 5′- AGCAGGGACCATAGCTCTCA-3′; Bnip3l-rv: 5′- TCATGGCTCCACTTTTCCTC-3′;

LC3-fw: 5′- CCTGAACTGAGCTGCCTCTAC-3′; LC3-rv: 5′- CCCAGAGGGACAACCCTAAC-3′;

ATF3-fw: 5′- GCTGCCAAGTGTCGAAACAAG-3′; ATF3-rv: 5′- CAGTTTTCCAATGGCTTCAGG-3′;

ATF4-fw: 5′- ATGGCCGGCTATGGATGAT-3′; ATF4-rv: 5′- CGAAGTCAAACTCTTTCAGATCCATT-3′;

SOD1-fw: 5′- CAAGCGGTGAACCAGTTGTG-3′; SOD1-rv: 5′- TGAGGTCCTGCACTGGTAC-3′;

SOD2-fw: 5′- GCCTGCACTGAAGTTCAATG-3′; SOD2-rv: 5′- ATCTGTAAGCGACCTTGCTC-3′;

Catalase-fw: 5′- ACCCTCTTATACCAGTTGGC-3′; Catalase-rv: 5′- GCATGCACATGGGGCCATCA-3′;

M-GPX1-fw: 5′- ACAGCCGCCGCTTTCGTACC-3′; M-GPX1-rv: 5′- ATGCCAGGGCCGCCTTAGGA-3′.

### Immunoblotting

Frozen muscles were pulverized by means of Qiagen Tissue Lyser and protein extracts were prepared in a buffer containing 50 mM Tris pH 7.5, 150 mM NaCl, 5 mM MgCl_2_, 1 mM DTT, 10% glycerol, 2% SDS, 1% Triton X-100, Roche Complete Protease Inhibitor Cocktail, 1 mM PMSF, 1 mM NaVO_3_, 5 mM NaF and 3 mM β- glycerophosphate and quantified using the BCA Protein Assay Kit (Thermo Scientific) following the manufacturer instructions. 40 µg of protein were separated by SDS-PAGE, transferred onto nitrocellulose membranes (Bio-Rad) and probed using the indicated antibodies: Beclin, LC3B (Cell Signaling); Actin (Santa Cruz).

### Protein carbonyls detection

Oxidation of muscle proteins was determined by detecting the presence of carbonyl groups. For this purpose the OxyBlot Protein Oxidation Detection Kit was used (Millipore). Briefly, the carbonyl groups in the protein side chains were derivatized to 2,4-dinitrophenylhydrazone (DNP-hydrazone) by reaction with 2,4-dinitrophenylhydrazine (DNPH). The DNP-derivatized protein samples were separated by polyacrylamide gel electrophoresis followed by Western blotting with primary antibody specific to the DNP moiety of the proteins. All values were normalized for the housekeeping protein Actin (Santa Cruz).

### Muscle ATP extraction and ATP quantification

ATP was extracted from frozen sections of TA muscles. Tissue was homogenized with 1 ml of ice-cold phenol-chloroform-isoamylalchol (25:24:1). 200 µl of PBS were added to the homogenate, and the latter was centrifuged at 14000 rpm for 10 min at 4 °C for phase separation. 20 µl of the upper aqueous phase was used to measure ATP. ATP measurements were performed with ATPlite Luminescence Assay System (Perkin Elmer) according to manufacturer’s instructions. Final results were normalized for muscle weight.

### Single myofibres culture

OCR measurements were carried out on freshly isolated FDB fibers. Muscles were digested in collagenase A (4 mg/ml) (Roche) dissolved in Tyrode’s salt solution (pH 7.4) (Sigma-Aldrich) containing 10% fetal bovine serum (Thermo Fisher Scientific). Isolated fibers were sedimented for 2 hours at 37 °C on laminin-coated XF24 microplate wells in DMEM (D5030 Sigma), supplemented with 1 mM NaPyr, 5 mM glucose, 33 mM NaCl, 15 mg phenol red, 25 mM HEPES, 10 mL di L-Glutamine.

### OCR (oxygen consumption rate) experiment

The rate of oxygen consumption was assessed in intact fibers using the XF24 Extracellular Flux Analyzer (Seahorse Biosciences), which allows the monitoring of OCR changes after up to four sequential additions of compounds (oligomycin 2 μM, FCCP 0.6 μM, rotenone 1 μM and antimycin A 1 μM). Fibers were prepared as reported above. A titration with the uncoupler FCCP was first performed, in order to optimize the FCCP concentration (0.6 μM) that maximally increases OCR. OCR measurements were normalized for the amount of fibers using Calcein as cellular marker. At the end of OCR measurements, fibers were loaded with Calcein-AM (2 μM, Sigma) for 20 min, and fluorescence (excitation 485/10 nm, emission 525/30 nm) was measured in well-scan mode using a Perkin Elmer EnVision plate reader.

### Statistical methods

Statistical data are presented as mean ± SEM; significance was calculated by t test or pairwise multiple comparison according to the number of independent factors present in each experiment, as detailed in the figure legends. *p < 0.05, **p < 0.01, ***p < 0.001. Raw data can be found as Supplementary Table S1.

## Electronic supplementary material

Supplementary information

## References

[1] Zuo L, Pannell BK (2015). Redox Characterization of Functioning Skeletal Muscle. Front. Physiol..

[2] Lo Verso F, Carnio S, Vainshtein A, Sandri M (2014). Autophagy is not required to sustain exercise and PRKAA1/AMPK activity but is important to prevent mitochondrial damage during physical activity. Autophagy.

[3] Pellegrino MA (2011). Redox homeostasis, oxidative stress and disuse muscle atrophy. J. Physiol..

[4] Cannavino J, Brocca L, Sandri M, Bottinelli R, Pellegrino MA (2014). PGC1-α over-expression prevents metabolic alterations and soleus muscle atrophy in hindlimb unloaded mice. J. Physiol..

[5] Migliaccio E (1999). The p66shc adaptor protein controls oxidative stress response and life span in mammals. Nature.

[6] Pinton P (2007). Protein Kinase C and Prolyl Isomerase 1 Regulate Mitochondrial Effects of the Life-Span Determinant p66Shc. Science (80-.)..

[7] Giorgio M (2005). Electron transfer between cytochrome c and p66Shc generates reactive oxygen species that trigger mitochondrial apoptosis. Cell.

[8] Carpi A (2009). The cardioprotective effects elicited by p66Shc ablation demonstrate the crucial role of mitochondrial ROS formation in ischemia/reperfusion injury. Biochim. Biophys. Acta - Bioenerg..

[9] Zaccagnini G (2007). p66ShcA and Oxidative Stress Modulate Myogenic Differentiation and Skeletal Muscle Regeneration after Hind Limb Ischemia. J. Biol. Chem..

[10] Xi G, Shen X, Radhakrishnan Y, Maile L, Clemmons D (2010). Hyperglycemia-induced p66shc inhibits insulin-like growth factor I-dependent cell survival via impairment of Src kinase-mediated phosphoinositide-3 kinase/AKT activation in vascular smooth muscle cells. Endocrinology.

[11] Pagnin E (2005). Diabetes Induces p66^shc^ Gene Expression in Human Peripheral Blood Mononuclear Cells: Relationship to Oxidative Stress. J. Clin. Endocrinol. Metab..

[12] Menini S (2007). Ablation of the gene encoding p66Shc protects mice against AGE-induced glomerulopathy by preventing oxidant-dependent tissue injury and further AGE accumulation. Diabetologia.

[13] Di Stefano V (2009). p66ShcA modulates oxidative stress and survival of endothelial progenitor cells in response to high glucose. Cardiovasc. Res..

[14] Berniakovich I (2008). p66Shc-generated Oxidative Signal Promotes Fat Accumulation. J. Biol. Chem..

[15] Tomilov, A. a. *et al*. The Shc locus regulates insulin signaling and adiposity in mammals. *Aging Cell* **10**, 55–65 (2011). 10.1111/j.1474-9726.2010.00641.x PMC4157392 21040401

[16] Fadini GP (2010). The redox enzyme p66Shc contributes to diabetes and ischemia-induced delay in cutaneous wound healing. Diabetes.

[17] Menini S (2006). Deletion of p66Shc longevity gene protects against experimental diabetic glomerulopathy by preventing diabetes-induced oxidative stress. Diabetes.

[18] Camici GG (2007). Genetic deletion of p66(Shc) adaptor protein prevents hyperglycemia-induced endothelial dysfunction and oxidative stress. Proc. Natl. Acad. Sci. USA.

[19] Rota M (2006). Diabetes promotes cardiac stem cell aging and heart failure, which are prevented by deletion of the p66shc gene. Circ. Res..

[20] Ranieri SC (2010). Mammalian life-span determinant p66shcA mediates obesity-induced insulin resistance. Proc. Natl. Acad. Sci..

[21] Ciciliot S (2015). p66Shc deletion or deficiency protects from obesity but not metabolic dysfunction in mice and humans. Diabetologia.

[22] Jensen, T. E. & Richter, E. a. Regulation of glucose and glycogen metabolism during and after exercise. *J. Physiol*. **590**, 1069–1076 (2012). 10.1113/jphysiol.2011.224972 PMC3381815 22199166

[23] Frøsig, C. & Richter, E. a. Improved Insulin Sensitivity After Exercise: Focus on Insulin Signaling. *Obesity* **17**, S15–S20 (2009). 10.1038/oby.2009.383 19927140

[24] Natalicchio A (2008). Involvement of the p66Shc protein in glucose transport regulation in skeletal muscle myoblasts. AJP Endocrinol. Metab..

[25] Zierath JR, Wallberg-Henriksson H (1992). Exercise training in obese diabetic patients. Special considerations. Sports Med..

[26] Pedersen BK, Febbraio MA (2012). Muscles, exercise and obesity: skeletal muscle as a secretory organ. Nat. Rev. Endocrinol..

[27] Wang, P., Li, C. G., Qi, Z., Cui, D. & Ding, S. Acute Exercise Induced Mitochondrial H 2 O 2 Production in Mouse Skeletal Muscle: Association with p 66Shc and FOXO3a Signaling and Antioxidant Enzymes. **2015** (2015). 10.1155/2015/536456 PMC4385701 25874020

[28] Schiaffino S, Reggiani C (2011). Fiber Types in Mammalian Skeletal Muscles. Physiol. Rev..

[29] Merry, T. L. & Ristow, M. Do antioxidant supplements interfere with skeletal muscle adaptation to exercise training? *J. Physiol*. **594**, 5135–47 (2015). 10.1113/JP270654 PMC5023714 26638792

[30] Furukawa S (2004). Increased oxidative stress in obesity and its impact on metabolic syndrome. J. Clin. Invest..

[31] Allwood, M. a. *et al*. Respiratory muscle weakness in the Zucker diabetic fatty rat. *Am. J. Physiol. - Regul. Integr. Comp. Physiol*. **309**, R780–7 (2015). 10.1152/ajpregu.00447.2014 26246509

[32] Sishi B (2011). Diet-induced obesity alters signalling pathways and induces atrophy and apoptosis in skeletal muscle in a prediabetic rat model. Exp. Physiol..

[33] Mammucari C (2007). FoxO3 Controls Autophagy in Skeletal Muscle *In Vivo*. Cell Metab..

[34] Grumati P (2011). Physical exercise stimulates autophagy in normal skeletal muscles but is detrimental for collagen VI-deficient muscles. Autophagy.

[35] He C (2012). Exercise-induced BCL2-regulated autophagy is required for muscle glucose homeostasis. Nature.

[36] Wu J (2011). The unfolded protein response mediates adaptation to exercise in skeletal muscle through a PGC-1α/ATF6α complex. Cell Metab..

[37] Wu J (2007). ATF6alpha optimizes long-term endoplasmic reticulum function to protect cells from chronic stress. Dev. Cell.

[38] Reczek CR, Chandel NS (2015). ROS-dependent signal transduction. Curr. Opin. Cell Biol..

[39] Mammucari C, Rizzuto R (2010). Signaling pathways in mitochondrial dysfunction and aging. Mech. Ageing Dev..

[40] Pereira Panza VSS, Diefenthaeler F, da Silva EL (2015). Benefits of dietary phytochemical supplementation on eccentric exercise-induced muscle damage: Is including antioxidants enough?. Nutrition.

[41] Blaauw B (2009). Inducible activation of Akt increases skeletal muscle mass and force without satellite cell activation. FASEB J..

[42] Schindelin J (2012). Fiji: an open-source platform for biological-image analysis. Nat. Methods.

[43] Aguiari P (2008). High glucose induces adipogenic differentiation of muscle-derived stem cells. Proc. Natl. Acad. Sci. USA.

[44] Blough ER, Rennie ER, Zhang F, Reiser PJ (1996). Enhanced electrophoretic separation and resolution of myosin heavy chains in mammalian and avian skeletal muscles. Anal. Biochem..

[45] Rozen S, Skaletsky H (2000). Primer3 on the WWW for general users and for biologist programmers. Methods Mol. Biol..

