# Supplementary material for: Role of p66shc in skeletal muscle function
Source: Sci Rep. 2017 Jul 24;7:6283. doi: 10.1038/s41598-017-06363-0 (PMC5524746; doi:10.1038/s41598-017-06363-0)
Supplement: Supplementary file 1 — Supplementary information [file 41598_2017_6363_MOESM1_ESM.pdf]

Supplementary information

Role of p66shc in skeletal muscle function

Veronica Granatiero, Gaia Gherardi, Matteo Vianello, Elsa Salerno, Erika Zecchini, Luana Toniolo, Giorgia Pallafacchina, Marta Murgia, Bert Blaauw, Rosario Rizzuto, Cristina Mammucari

Supplementary Figure S1

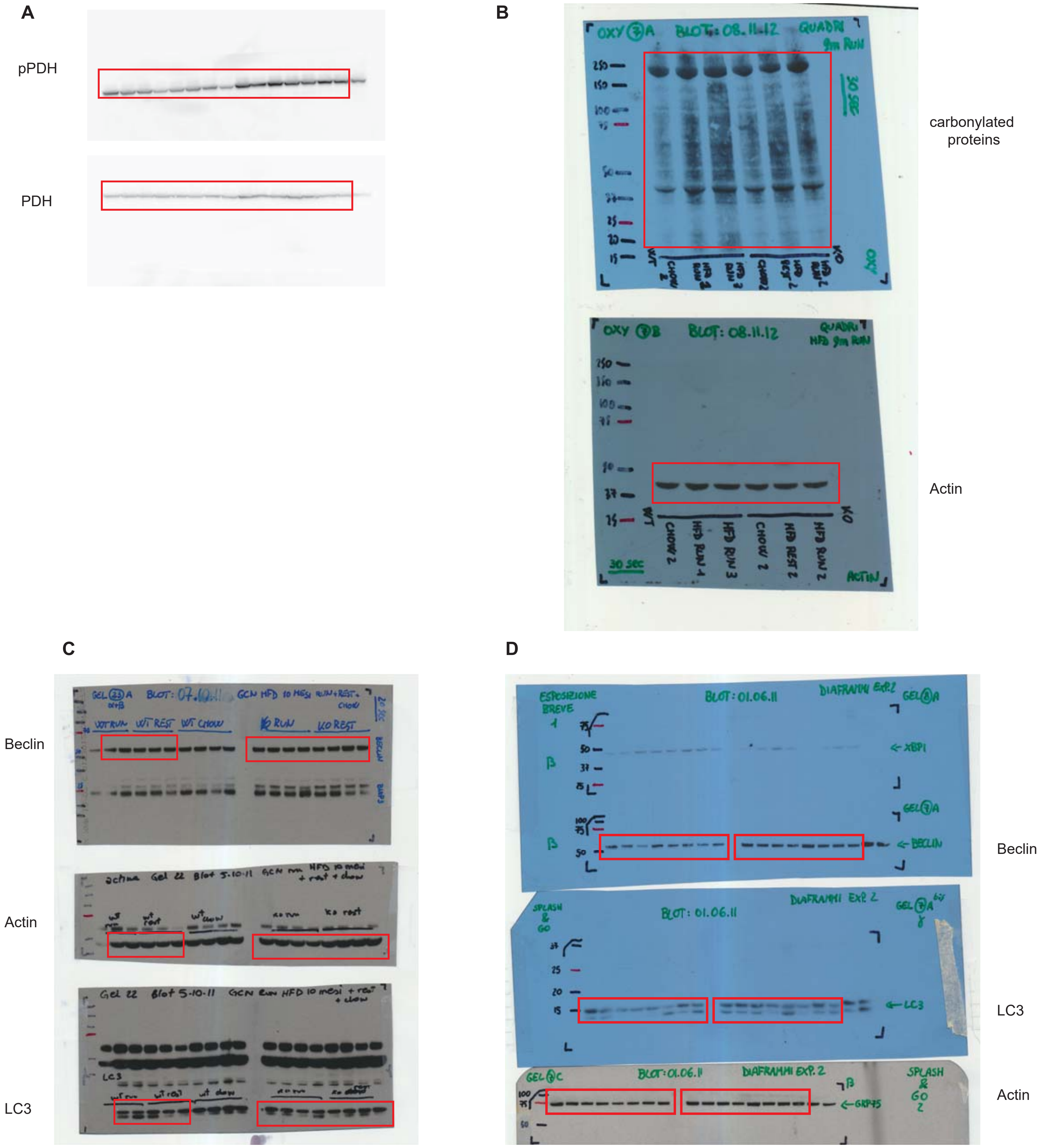

Supplementary Table S1

Experiment	Measurement	Mean ± SEM	Figure
**Wt**	Fibre size (μm^2)	2258.262 ± 242.466	1B
**p66shc -/-**	Fibre size (μm^2)	2174.585 ± 288.611	1B
**Wt**	mRNA level (p66shc/GAPDH)	1 ± 0.13	1C
**Wt + den**	mRNA level (p66shc/GAPDH)	5.29 ± 0.83	1C
**Wt**	Fibre size (μm^2)	2581.5 ± 256.1	1D
**Wt + den**	Fibre size (μm^2)	1660.1 ± 189.2	1D
**p66shc -/-**	Fibre size (μm^2)	2555.3 ± 166.7	1D
**p66shc -/- + den**	Fibre size (μm^2)	1598.3 ± 87.2	1D
**Wt**	% of myofibers	0.16	1E
**Wt + den**	% of myofibers	1.38	1E
**p66shc -/-**	% of myofibers	0.7	1E
**p66shc -/- + den**	% of myofibers	2.18	1E
**Wt**	% of myofibers	9.71	1E
**Wt + den**	% of myofibers	21.84	1E
**p66shc -/-**	% of myofibers	12.08	1E
**p66shc -/- + den**	% of myofibers	26.67	1E
**Wt**	% of myofibers	21.47	1E
**Wt + den**	% of myofibers	36.42	1E
**p66shc -/-**	% of myofibers	19.64	1E
**p66shc -/- + den**	% of myofibers	30.07	1E
**Wt**	% of myofibers	18.5	1E
**Wt + den**	% of myofibers	29.52	1E
**p66shc -/-**	% of myofibers	15.24	1E
**p66shc -/- + den**	% of myofibers	25.63	1E
**Wt**	% of myofibers	16.72	1E
**Wt + den**	% of myofibers	9.34	1E
**p66shc -/-**	% of myofibers	16.22	1E
**p66shc -/- + den**	% of myofibers	12.99	1E
**Wt**	% of myofibers	17.4	1E
**Wt + den**	% of myofibers	1.3	1E
**p66shc -/-**	% of myofibers	17.68	1E
**p66shc -/- + den**	% of myofibers	2.15	1E
**Wt**	% of myofibers	10.52	1E
**Wt + den**	% of myofibers	0.17	1E
**p66shc -/-**	% of myofibers	13.22	1E
**p66shc -/- + den**	% of myofibers	0.28	1E
**Wt**	% of myofibers	4.15	1E
**Wt + den**	% of myofibers	0.02	1E
**p66shc -/-**	% of myofibers	4.14	1E
**p66shc -/- + den**	% of myofibers	0.03	1E
**Wt**	% of myofibers	1.37	1E
**Wt + den**	% of myofibers	0	1E
**p66shc -/-**	% of myofibers	1.08	1E
**p66shc -/- + den**	% of myofibers	0.01	1E
**Wt**	% 2b MyHC	74.8 ± 5.9	1F
**Wt**	% 2x MyHC	24.2 ± 5.9	1F
**p66shc -/-**	% 2b MyHC	73.5 ± 4.7	1F
**p66shc -/-**	% 2x MyHC	25.5 ± 4.8	1F
**TA Wt**	mRNA level	1 ± 0.04	1G

Supplementary Table S1

	(p66shc/GAPDH)		
**EDL Wt**	mRNA level (p66shc/GAPDH)	1.16 ± 0.29	1G
**SOL Wt**	mRNA level (p66shc/GAPDH)	0.88 ± 0.15	1G
**Wt**	ng glycogen/ mg muscle	6.94 ± 1.33	2C
**p66shc -/-**	ng glycogen/ mg muscle	6.95 ± 1.51	2C
**Wt EDL**	Normalized intensity (pPDH/PDH)	1 ± 0.13	2D
**p66shc -/- EDL**	Normalized intensity (pPDH/PDH)	0.85 ± 0.10	2D
**Wt SOL**	Normalized intensity (pPDH/PDH)	1 ± 0.05	2D
**p66shc -/- SOL**	Normalized intensity (pPDH/PDH)	1.20 ± 0.13	2D
**Wt**	OCR (pmol/min)	716 ± 90	2E
**Wt**	OCR (pmol/min)	735 ± 65	2E
**Wt**	OCR (pmol/min)	719 ± 68	2E
**Wt**	OCR (pmol/min)	322 ± 41	2E
**Wt**	OCR (pmol/min)	340 ± 34	2E
**Wt**	OCR (pmol/min)	381 ± 30	2E
**Wt**	OCR (pmol/min)	638 ± 66	2E
**Wt**	OCR (pmol/min)	618 ± 47	2E
**Wt**	OCR (pmol/min)	633 ± 43	2E
**Wt**	OCR (pmol/min)	226 ± 24	2E
**Wt**	OCR (pmol/min)	216 ± 21	2E
**Wt**	OCR (pmol/min)	222 ± 21	2E
**Wt**	OCR (pmol/min)	193 ± 16	2E
**Wt**	OCR (pmol/min)	186 ± 16	2E
**Wt**	OCR (pmol/min)	179 ± 15	2E
**p66shc -/-**	OCR (pmol/min)	623 ± 85	2E
**p66shc -/-**	OCR (pmol/min)	643 ± 64	2E
**p66shc -/-**	OCR (pmol/min)	674 ± 40	2E
**p66shc -/-**	OCR (pmol/min)	343 ± 44	2E
**p66shc -/-**	OCR (pmol/min)	321 ± 44	2E
**p66shc -/-**	OCR (pmol/min)	358 ± 41	2E
**p66shc -/-**	OCR (pmol/min)	684 ± 71	2E
**p66shc -/-**	OCR (pmol/min)	650 ± 71	2E
**p66shc -/-**	OCR (pmol/min)	663 ± 85	2E
**p66shc -/-**	OCR (pmol/min)	236 ± 22	2E
**p66shc -/-**	OCR (pmol/min)	222 ± 20	2E
**p66shc -/-**	OCR (pmol/min)	217 ± 14	2E
**p66shc -/-**	OCR (pmol/min)	200 ± 13	2E
**p66shc -/-**	OCR (pmol/min)	192 ± 12	2E
**p66shc -/-**	OCR (pmol/min)	176 ± 14	2E
**Wt**	Relative muscle ATP	1 ± 0.30	2F
**p66shc -/-**	Relative muscle ATP	1.16 ± 0.29	2F
**Wt**	Running distance (meters)	1284 ± 273	3A
**p66shc -/-**	Running distance (meters)	1158 ± 220	3A
**Wt 1° day**	Running distance (meters)	1441.2 ± 172.7	3B
**Wt 2° day**	Running distance (meters)	1428.7 ± 186.8	3B
**Wt 3° day**	Running distance (meters)	1473.5 ± 181.9	3B

Supplementary Table S1

**p66shc -/- 1° day**	Running distance (meters)	1617.9 ± 113.3	3B
**p66shc -/- 2° day**	Running distance (meters)	1430.5 ± 100.6	3B
**p66shc -/- 3° day**	Running distance (meters)	1289 ± 85.7	3B
**Wt 0 mo HFD**	Weight (g)	20.8 ± 1	4A
**Wt 4 mo HFD**	Weight (g)	28.8 ± 1.4	4A
**Wt 9 mo HFD**	Weight (g)	35.4 ± 2.3	4A
**p66shc -/- 0 mo HFD**	Weight (g)	17.4 ± 0.9	4A
**p66shc -/- 4 mo HFD**	Weight (g)	23.5 ± 0.8	4A
**p66shc -/- 9 mo HFD**	Weight (g)	26.3 ± 1.2	4A
**Wt**	Carbonylated proteins	1	4B
**Wt HFD 1**	Carbonylated proteins	2.23	4B
**Wt HFD 2**	Carbonylated proteins	2.99	4B
**p66shc -/-**	Carbonylated proteins	1.74	4B
**p66shc -/- HFD 1**	Carbonylated proteins	2.34	4B
**p66shc -/- HFD 2**	Carbonylated proteins	2.09	4B
**Wt**	Weight (mg)	125.3 ± 7.94	4C
**p66shc -/-**	Weight (mg)	140 ± 9.98	4C
**Wt + HFD**	Weight (mg)	150.9 ± 6.02	4C
**p66shc -/- + HFD**	Weight (mg)	135.7 ± 5.01	4C
**Wt**	Fibre size (μm^2)	2258.3 ± 242.5	4D
**p66shc -/-**	Fibre size (μm^2)	2174.6 ± 288.6	4D
**Wt**	% 2b MyHC	79 ± 4	4E
**Wt**	% 2x MyHC	18 ± 3	4E
**p66shc -/-**	% 2b MyHC	63 ± 9	4E
**p66shc -/-**	% 2x MyHC	27 ± 5	4E
**Wt**	ng glycogen/ mg muscle	6.68 ± 0.67	4J
**p66shc -/-**	ng glycogen/ mg muscle	6.32 ± 0.58	4J
**Wt 0 mo HFD**	Running distance (meters)	1284 ± 237	5A
**Wt 4 mo HFD**	Running distance (meters)	870 ± 110	5A
**Wt 9 mo HFD**	Running distance (meters)	407 ± 669	5A
**p66shc -/- 0 mo HFD**	Running distance (meters)	1158 ± 220	5A
**p66shc -/- 4 mo HFD**	Running distance (meters)	1232 ± 686	5A
**p66shc -/- 9 mo HFD**	Running distance (meters)	945 ± 158	5A
**Wt**	Muscle force (N/Kg)	35234 ± 877	5B
**p66shc -/-**	Muscle force (N/Kg)	35382 ± 2072	5B
**Wt + HFD**	Muscle force (N/Kg)	34721 ± 1001	5B
**p66shc -/- + HFD**	Muscle force (N/Kg)	34867 ± 893	5B
**Wt**	Muscle force (N/Kg)	34721 ± 1001	5C
**p66shc -/-**	Muscle force (N/Kg)	34866 ± 893	5C
**Wt + exercise**	Muscle force (N/Kg)	30282 ± 1316	5C
**p66shc -/- + exercise**	Muscle force (N/Kg)	36204 ± 1084	5C
**Wt**	Normalized intensity (LC3II/actin)	1 ± 0.39	6A
**p66shc -/-**	Normalized intensity (LC3II/actin)	2.51 ± 0.44	6A
**Wt + exercise**	Normalized intensity	2.54 ± 0.72	6A

Supplementary Table S1

	(LC3II/actin)		
**p66shc -/- + exercise**	Normalized intensity (LC3II/actin)	2.58 ± 0.37	6A
**Wt**	Normalized intensity (beclin/actin)	1 ± 0.18	6A
**p66shc -/-**	Normalized intensity (beclin/actin)	1.41 ± 0.12	6A
**Wt + exercise**	Normalized intensity (beclin/actin)	0.97 ± 0.06	6A
**p66shc -/- + exercise**	Normalized intensity (beclin/actin)	1.23 ± 0.06	6A
**Wt**	mRNA level (BNIP3/GAPDH)	1 ± 0.24	6B
**p66shc -/-**	mRNA level (BNIP3/GAPDH)	1.12 ± 0.31	6B
**Wt + exercise**	mRNA level (BNIP3/GAPDH)	1.89 ± 0.19	6B
**p66shc -/- + exercise**	mRNA level (BNIP3/GAPDH)	1.68 ± 0.32	6B
**Wt**	mRNA level (BNIP3l/GAPDH)	1 ± 0.45	6C
**p66shc -/-**	mRNA level (BNIP3l/GAPDH)	1.23 ± 0.16	6C
**Wt + exercise**	mRNA level (BNIP3l/GAPDH)	1.77 ± 0.33	6C
**p66shc -/- + exercise**	mRNA level (BNIP3l/GAPDH)	1.80 ± 0.17	6C
**Wt**	mRNA level (ATF3/GAPDH)	1 ± 0.2	6D
**p66shc -/-**	mRNA level (ATF3/GAPDH)	0.59 ± 0.09	6D
**Wt + exercise**	mRNA level (ATF3/GAPDH)	5 ± 1.93	6D
**p66shc -/- + exercise**	mRNA level (ATF3/GAPDH)	5.03 ± 1.34	6D
**Wt**	mRNA level (ATF4/GAPDH)	1 ± 0.21	6E
**p66shc -/-**	mRNA level (ATF4/GAPDH)	0.94 ± 0.09	6E
**Wt + exercise**	mRNA level (ATF4/GAPDH)	2.07 ± 0.75	6E
**p66shc -/- + exercise**	mRNA level (ATF4/GAPDH)	2.35 ± 0.82	6E
**Wt**	Normalized intensity (LC3II/actin)	1 ± 0.56	7A
**p66shc -/-**	Normalized intensity (LC3II/actin)	0.54 ± 0.17	7A
**Wt + exercise**	Normalized intensity (LC3II/actin)	2.14 ± 0.42	7A
**p66shc -/- + exercise**	Normalized intensity (LC3II/actin)	1.07 ± 0.30	7A
**Wt**	Normalized intensity (beclin/actin)	1 ± 0.05	7A
**p66shc -/-**	Normalized intensity (beclin/actin)	0.89 ± 0.03	7A
**Wt + exercise**	Normalized intensity	0.87 ± 0.13	7A

Supplementary Table S1

	(beclin/actin)		
**p66shc -/- + exercise**	Normalized intensity (beclin/actin)	1.06 ± 0.05	7A
**Wt**	mRNA level (BNIP3/GAPDH)	1 ± 0.2	7B
**p66shc -/-**	mRNA level (BNIP3/GAPDH)	0.77 ± 0.05	7B
**Wt + exercise**	mRNA level (BNIP3/GAPDH)	0.78 ± 0.17	7B
**p66shc -/- + exercise**	mRNA level (BNIP3/GAPDH)	0.84 ± 0.19	7B
**Wt**	mRNA level (BNIP3l/GAPDH)	1 ± 0.39	7C
**p66shc -/-**	mRNA level (BNIP3l/GAPDH)	1.14 ± 0.31	7C
**Wt + exercise**	mRNA level (BNIP3l/GAPDH)	0.96 ± 0.12	7C
**p66shc -/- + exercise**	mRNA level (BNIP3l/GAPDH)	1.32 ± 0.22	7C
**Wt**	mRNA level (LC3/GAPDH)	1 ± 0.34	7D
**p66shc -/-**	mRNA level (LC3/GAPDH)	0.80 ± 0.38	7D
**Wt + exercise**	mRNA level (LC3/GAPDH)	0.85 ± 0.16	7D
**p66shc -/- + exercise**	mRNA level (LC3/GAPDH)	1.08 ± 0.34	7D
**Wt**	mRNA level (ATF3/GAPDH)	1 ± 0.22	7E
**p66shc -/-**	mRNA level (ATF3/GAPDH)	0.29 ± 0.14	7E
**Wt + exercise**	mRNA level (ATF3/GAPDH)	3.52 ± 0.26	7E
**p66shc -/- + exercise**	mRNA level (ATF3/GAPDH)	1.56 ± 0.29	7E
**Wt**	mRNA level (SOD1/GAPDH)	1 ± 0.19	7F
**p66shc -/-**	mRNA level (SOD1/GAPDH)	0.93 ± 0.27	7F
**Wt + exercise**	mRNA level (SOD1/GAPDH)	0.80 ± 0.04	7F
**p66shc -/- + exercise**	mRNA level (SOD1/GAPDH)	1 ± 0.19	7F
**Wt**	mRNA level (SOD2/GAPDH)	1 ± 0.19	7G
**p66shc -/-**	mRNA level (SOD2/GAPDH)	0.78 ± 0.30	7G
**Wt + exercise**	mRNA level (SOD2/GAPDH)	1.10 ± 0.17	7G
**p66shc -/- + exercise**	mRNA level (SOD2/GAPDH)	1.27 ± 0.15	7G
**Wt**	mRNA level (Catalase/GAPDH)	1 ± 0.27	7H
**p66shc -/-**	mRNA level (Catalase/GAPDH)	1.09 ± 0.23	7H
**Wt + exercise**	mRNA level (Catalase/GAPDH)	0.85 ± 0.18	7H
**p66shc -/- + exercise**	mRNA level (Catalase/GAPDH)	1.04 ± 0.14	7H
**Wt**	mRNA level	1 ± 0.18	7I

Supplementary Table S1

	(M-GPX1/GAPDH)		
**p66shc -/-**	mRNA level (M-GPX1/GAPDH)	1.18 ± 0.22	7I
**Wt + exercise**	mRNA level (M-GPX1/GAPDH)	1.04 ± 0.16	7I
**p66shc -/- + exercise**	mRNA level (M-GPX1/GAPDH)	0.83 ± 0.03	7I

Supplementary Figure and Table legends.

Supplementary Figure S1. Full-length blots related to the cropped blots presented in the main figures.

A Full-length blots relative to Figure 2D.
B Full-length blots relative to Figure 4B.
C Full-length blots relative to Figure 6A.
D Full-length blots relative to Figure 7A.

Supplementary Table S1. Row data related to main figures.

For each experiment, the table reports name, type, mean value +/- SEM and relative Figure.
